# New Curriculum of the Apothecaries' Company, with Explanations and Comments

**Published:** 1829-01-01

**Authors:** 


### NEW CURRICULUM OF THE APOTHECARIES' COMPANY.

It has been somewhere observed, that the *laws* of England appear to have been framed for the purpose of creating law-suits. The laws and regulations emanating from Apothecaries' Hall, flow with such rapidity upon the profession, we hardly can tell where the one begins or the other ends. Thus, there was a curriculum issued in December, 1827, which was not to be operative till February, 1828. This code had hardly begun to be acted on at all, when, in September, 1828, out comes a new regulation superseding its predecessor, and so worded, that not one student in ten can make out its meaning! Here it is.

"Every canditate for a certificate to practise as an Apothecary will be required to possess a competent knowledge of the Latin language; and in compliance with the 14th and 15th sections of the said Act, to produce testimonials of having served an apprenticeship of not less than five years to an Apothecary; of having attained the full age of 21 years, and being of good moral conduct.

"He will also be required to produce certificates of having attended not less than—

"Two Courses of Lectures on Chemistry;

"Two Courses of Lectures on Materia Medica and Botany;

"Two Courses of Lectures on Anatomy and Physiology;

"Two Courses of Anatomical Demonstrations;

"Two Courses of Lectures on the Theory and Practice of Medicine—these last to be attended subsequently to one Course of Lectures on Materia Medica, Chemistry, and Anatomy;

"And a certificate* of attendance for six months,† at least, on the Physicians' Practice of some public Hospital or Infirmary (containing not less than sixty beds), or for nine months at a Dispensary—such attendance to commence subsequently to the first Course of Lectures on the Principles and Practice of Medicine.

"The regulations relating to the order of succession in which the Lectures on the Practice of Medicine, and the Physicians' Practice of an Hospital or Dispensary, are to be attended, are designed to apply to those Students only who commenced their attendance on Lectures on or after the 1st of February, 1828; and all such persons are particularly requested to take notice, that unless they shall have strictly complied with such order of succession, they will not be admitted to an examination.

"In addition to the course of study above required as indispensably necessary, candidates are earnestly recommended to attend Clinical Lectures, and also Lectures on Midwifery, and the Diseases of Women and Children, on the latter of which subjects, as an important part of Medical Practice, they will be examined.

"The court have determined that the examination of the candidate shall be as follows:—

"1. In translating grammatically parts of the Pharmacopœia Londinensis, and Physicians' Prescriptions; and after the 1st of January, 1831, candidates will be required to translate portions of the following Medical Latin Authors, viz. Celsus de Medicina, or Gregory's Conspectus Medicinæ Theoreticæ.

"2. In Chemistry.

"3. In the Materia Medica.

"4. In Botany.

"5. In Anatomy and Physiology.

"6. In the Practice of Medicine."

We pass over the inconsistency of ma-

---

* "Physicians' Pupils who intend to present themselves for examination, must appear personally at the Beadle's Office, in this Hall, and bring with them the tickets authorizing their attendance on such practice, as the commencement thereof will be dated from the time of such personal appearance."

† "All candidates applying for examination after the 1st of October, 1829, will be required to produce evidence of having attended the Physicians' Practice at an Hospital or Infirmary for nine months, or at a Dispensary for twelve months."

king two laws actually commence their operation at one period—28th of February, 1828, though the second is not promulgated till the September following. We are friendly to every regulation which increases the period of instruction—we mean *real instruction*—not worse than the annihilation of five precious years behind the counter! The regulation above-mentioned does not specify, whether the five years' apprenticeship must *precede* the attendance on lectures, or whether the attendance may be included within the period of apprenticeship. This point should be explained by the Company. But, while the system of apprenticeship is allowed to remain, all other improvements are of comparatively little avail. It is in vain for the Apothecaries' Company to talk about Acts of Parliament. It is as easy for them to direct the last three years of the apprenticeship to be spent in attendance on lectures, as the three years that succeed the indenture.

In respect to the foregoing curriculum, it is asked (more easily than solved) how long will it take to comply with the regulations? *We* say, one year, or one year and a quarter of *continuous* lectures. If the student studies only in Winter, it will require two or three years to complete the curriculum. Thus, one course, or three months of materia medica, chemistry, and anatomy, must be gone through, before the theory and practice of medicine is commenced, two courses of which will require six months, and thus, the student will be carried on till the end of June, (allowing that he began on the 1st of October)—provided he can get lectures on medicine in the middle of Summer. Six or nine months hospital attendance is to commence after the first course of medicine, say on the 1st of May, which will carry him on till the middle or end of October, in the one case, or till January in the other. Once more we implore the Company to do away with a portion of the mischief resulting from the indenture system, by directing, at least, half of the five years to be occupied with professional and scientific instruction, instead of the mechanical manipulations of the shop.

*Note.*—Mr. Watson has apprised the public, that the *last* regulation is only operative on those students who commenced their studies in *October* of the present year, and from whom *six* months, at least, of hospital, or *nine* of dispensary time will be expected. All who commence their studies in the *second course* of the present session, must produce *nine* months hospital, or *twelve* months dispensary time.

---

